# Atherogenic Index of Plasma and High-Sensitivity C-Reactive Protein Levels Among People Living With HIV on Dolutegravir and Ritonavir-Boosted Atazanavir-Based Antiretroviral Therapy and Their Correlations to CD4 Cell Counts

**DOI:** 10.1155/arat/1468678

**Published:** 2025-01-13

**Authors:** Nuredin Chura Waritu, Rashed Edris Usure, Bulcha Guye Adema, Mamud Umer Wakeyo, Mohammed Jemal

**Affiliations:** ^1^Department of Biomedical Sciences, School of Medicine, Wolaita Sodo University, Wolaita Sodo, Ethiopia; ^2^Department of Pharmaceutical Chemistry, School of Pharmacy, Hawassa University, Hawassa, Ethiopia; ^3^Department of Paediatrics, School of Nursing, Wolaita Sodo University, Wolaita Sodo, Ethiopia; ^4^Department of Epidemiology and Biostatistics, School of Public Health, Wolaita Sodo University, Wolaita Sodo, Ethiopia; ^5^Department of Biomedical Sciences, School of Medicine, Debre Markos University, Debre Markos, Ethiopia

**Keywords:** antiretroviral therapy, atherogenic index of plasma, dolutegravir, high-sensitivity C-reactive protein, ritonavir-boosted atazanavir

## Abstract

**Background:** Atherogenic index of plasma (AIP) and high-sensitivity C-reactive protein (hsCRP) levels which are strong predictors of the risk of cardiovascular disease (CVD) seen elevated in the serum of people living with HIV (PLWH) on HAART and in those with low cluster of differentiation-4 (CD4) cell counts. Thus, this study aimed to evaluate AIP and hsCRP levels among PLWH on dolutegravir (DTG) and ritonavir-boosted atazanavir-based (ATV/r) antiretroviral therapy (ART) and their correlations to CD4 cell counts.

**Methods:** The study design was an institutional-based comparative cross-sectional study conducted from November 4, 2021, to January 4, 2022. The total sample size was 172 with equal number of DTG and ritonavir-boosted atazanavir-treated PLWH. Participants were recruited by a consecutive sampling method. The data were entered into EpiData Version 4.6, then exported to SPSS Version 25.0, and analyzed using Chi-square, Student's *t*-test, and Pearson correlation. Statistical significance was set at *p* < 0.05.

**Results:** AIP was significantly higher among individuals on ATV/r, with levels in the range of 0.1–0.24 at 44.2% and > 0.24 at 31.4%, compared with those on DTG-based regimens of ART, which showed levels of 39.5% and 9.3%, respectively. Similarly, the higher hsCRP level of ≥ 2 mg/L was observed among patients on ATV/r (44.2%) than in DTG-based (24.4%) regimens of ART. AIP and hsCRP were negatively correlated with CD4 cell counts with Pearson correlation coefficients of −0.46 and −0.38, respectively.

**Conclusion:** From the study conducted, it can be concluded that the higher levels of AIP and hsCRP were seen in patients treated by ATV/r than in DTG-based regimens of ART and in PLWH with low CD4 cell counts. Therefore, routine monitoring of both AIP and hsCRP levels was a good marker of HIV disease progression and cardiovascular disease risk assessment in PLWH, particularly in developing countries where CD4 cell count testing is expensive and not easily available.

## 1. Background

Antiretroviral therapy (ART) improves the quality of life for those living with HIV by changing the normal course of the virus from one that is acutely fatal to one that is chronically manageable [1, 2]. Notwithstanding these advantages, there is growing worry over the risk of atherosclerotic cardiovascular disease (ASCVD) linked to HIV infection, which ART may exacerbate it further [3–6]. People living with HIV (PLWH) are more likely to develop cardiovascular disease (CVD) if they live longer with the virus and are exposed to ART for a longer duration [7]. Along with aberrant lipid profiles, chronic immunological activation that may the cause for elevated inflammation also linked to HIV and its therapy, were among the leading risk factors contributed to CVD [8].

Studies reported that increased total cholesterol (TC) and triglyceride (TG), low density lipoprotein cholesterol (LDL-C), and reduced levels of high density lipoprotein cholesterol (HDL-C) were increasingly seen among PLWH on ART [9, 10]. Nowadays, researchers stress on lipid ratios to predict the risk of CVD [11]. The atherogenic index of plasma (AIP), defined by the logarithm of the ratio of TG to HDL-C, is a critical index to predict the risk of CVD, for it is an independent strong predictive factor for rapid plaque progression and association with the level of LDL, TC/HDL, and LDL/HDL ratios [12–14].

There are evidences from several research studies indicating elevated levels of AIP among ART-treated PLWH among PLWH [15–17]. People with HIV with a low cluster of differentiation-4 (CD4) count have been found to have increased AIPs in the high-risk category [18]. AIP could be a more useful tool for doctors as a sensitive predictor for CVD if individual lipid parameter values were normal and for those with low CD4 cell counts [19].

Studies have shown that dolutegravir (DTG) is linked with better lipid profiles [20, 21]. Those with HIV who transitioned to DTG-based ART showed a lower median TG and mean level of AIP [22]. However, research from Brazil showed increased levels of TC and LDL-C among PLWH treated by DTG-based ART and the other from Uganda also reported increased LDL, TC, and TG and decreased levels of HDL among HIV patients on DTG-based ART [23, 24]. Most of the switch studies have complained that derangement in lipid profiles were common among PLWH treated by protease inhibitor (PI)-based ART and it was improved in patients switched from PI to integrase strand transfer inhibitor (ISTI)-containing regimens [25–27]. Studies demonstrated that unboosted-atazanavir is the safest ART concerning dyslipidemia and is associated with favorable lipid profile levels; however, this favorability was lost when boosting it by ritonavir [27]. Ritonavir-boosted atazanavir have been associated with increased TG and AIP and reduced levels of HDL-C [28–30].

Chronic immune activations and higher plasma levels of the inflammatory biomarkers were frequently seen in treatment-naive PLWH compared with ART-treated PLWH [31]. However, the other studies claim that higher high sensitivity C-reactive protein (hsCRP) levels and its association with traditional CVD risk factors in ART-treated PLWH constitutes importance of hsCRP as a marker for CVD risk assessment [30, 32, 33]. Accordingly, one study from Spain disclosed that C-reactive protein (CRP) is associated with traditional CVD risk factors so that in may be used as a marker for CVD risk related to HIV and its treatment [34].

There has been a concern that taking PI may lead to increased inflammation with increased markers of inflammations like hsCRP [25, 30, 33]. Switching patients from boosted PI to DTG have been associated with reduced monocyte activation and inflammation [35]. In contrary, some studies complain the association of INSTI with increased inflammation as measured by different biomarkers of inflammation including hsCRP levels [36, 37].

C-reactive protein is acute phase protein which is increasingly seen in PLWH as a result of opportunistic infections [38]. With progression to HIV, PLWH develops different types of opportunistic infections due to decreased T-lymphocytes with subsequent decreased in CD4 cell counts [39]. Thus, the CRP level increases with increase in opportunistic infection that makes CRP a better diagnostic biomarker that may reflect the immune suppression of the host [40].

In Ethiopia, most of the patients are on regimen containing DTG and ritonavir-boosted atazanavir (ATV/r). Specially DTG is a new INSTI and is effective in viral suppression has been recommended by the World Health Organization (WHO) for PLWH [41–43]. For developing countries like Ethiopia, cost-effective diagnostic biomarker is very important to follow the progress of HIV, ART response, and risk assessment for CVD. Due to the fact that CD4 cell count is not affordable in larger populations of Ethiopia, both hsCRP and AIP can be the most important biomarkers to monitor the degree of immune suppressions and their risk of developing CVD. The national as well as WHO ART guidelines do not include hsCRP and AIP to monitor the risk of CVD and the degree of immune suppression along with disease progression. In Ethiopia, few studies were done on AIP, hsCRP levels, and their correlation to CD4 cell count among PLWH on DTG- and ATV/r-based ART. Therefore, this study aimed to assess the serum AIP and hsCRP levels among PLWH on DTG- and ATV/r-based ART and their correlation to CD4 cell counts at Jimma University Medical Center, Southwest Ethiopia.

## 2. Methods and Materials

### 2.1. Study Setting and Period

The research was carried out at Jimma University Medical Center (JUMC) ART clinic. It is southwest Ethiopia's major referral hospital. Services are offered to residents of the town and the neighboring districts by JUMC ART clinic. On October 15, 2021, there were 3500 HIV-positive individuals who were active. The research was carried out between November 4, 2021, and January 4, 2022.

### 2.2. Study Design

The preferred design of this study was an institutional-based comparative cross-sectional study method.

### 2.3. Study Participant and Study Processes

In this study, PLWH age ≥ 18 years treated by DTG+3TC + TDF and ATV/r+3TC + TDF for at least six months [44] as well as who volunteered to participate were included. While patients were treated by DTG+3TC + TDF and ATV/r+3TC + TDF regimens for less than 6 months, patients with known diabetes mellitus and pregnancy due to metabolic changes [45, 46], patients with pre-existing liver and renal problems, active cancer, patients on antituberculosis drugs [47–50], patients taking lipid-lowering drugs, and who did not volunteer to participate were excluded.

### 2.4. Sample Size Determinations and Sampling Procedure

G^∗^ power statistical analysis Version 3.1 software was used to calculate sample size. It was calculated by using *α* = 0.05, power (1–*β*) = 90%, with a DTG to ATV/r ratio of 1:1, a two independent groups two tail *t*-test, and effect size = 0.5. Then, it gives the total sample size of 172. From this, 86 were DTG-based group and 86 were ATV/r-based group. Participants were selected by the consecutive sampling technique.

### 2.5. Data Collection Procedures

Two senior nurses and a laboratory technologist were trained on the purpose, methods, and ethical considerations of the study. The nurses then used a structured questionnaire to gather clinical and sociodemographic data, and the laboratory technologists took a sample of venous blood from each study participant to measure CD4 cell counts, hsCRP levels, and lipid profiles. Trained nurses used an SECA meter and a standard balance to get measurements. The person's weight in kilograms divided by the square of their height in meters (kg/m^2^) yielded their BMI.

### 2.6. Blood Sample Collection

For the measurements of lipid profiles, hsCRP, and CD4 cell count, 5 mL of blood was taken from the study participants. Whole blood was used to measure the CD4 cell count. The remaining blood was centrifuged and the serum obtained was used to determine lipid profiles and hsCRP levels.

### 2.7. Definitions

#### 2.7.1. AIP

AIP is a logarithmically transformed ratio of TG to HDL-c and is calculated using Log10 (TG/HDL-c) ratio. AIP is categorized according to its risk to CVD: < 0.1; low risk 0.1–0.24; medium risk > 0.24; and high risk [19].

#### 2.7.2. Elevated Serum hsCRP Levels

Patients having serum hsCRP levels ≥ 2 mg/L for both genders. It was categorized based on its risk enhancing factor for ASCVD [51].

### 2.8. Statistical Methods

The data collected were marked, entered, and cleaned using EpiData software Version 3.1 and then exported to and analyzed by SPSS Version 25. Descriptive statistics such as number, percentages, mean, and standard deviations were used to describe the variables. Chi-square and Student's *t*-test were used to detect differences between the groups. Pearson correlation was done to know the relation of AIP and hsCRP with CD4 cell counts. The level of significance for statistical analysis was set at a *p* value < 0.05.

### 2.9. Ethical Consideration

The Jimma University Institutional Review Board provided ethical clearance and approved the study methodology (ref: IHRPG1/5/2021). All patients provided written informed consent following the interview. To maintain participant confidentiality, information about them was coded and kept private while data were analyzed. The Helsinki Declaration was followed in the conduct of this investigation.

## 3. Results

### 3.1. Sociodemographic Characteristics

From the total 172 of our study participants, 86 were on DTG-based regimens and 86 were on ATV/r-based regimens. Patients in both DTG- and ATV/r-based regimen groups had a comparable proportion of age ≥ 40, as described in Table 1. Regarding gender, 66.3% and 69.8% of DTG were females. For others, sociodemographic characteristics also showed a nearly comparable difference.

### 3.2. Clinical Characteristics of the Study Participants

From our study result, it was seen that the higher but insignificant mean level of CD4 cell count was detected among PLWH on DTG (615 cells/mm^3^) compared with ATV/r-based regimens of ART (543 cells/mm^3^). Patients taking ART for longer duration accounts 58.1% for DTG-based and 47.7% for ATV/r-based regimens of ART. Patients living with HIV for > 5 years were observed to be 46.5% (40/86) and 43% (37/86) among patients on DTG- and ATV/r-based regimens, respectively. Regarding BMI, the insignificant higher level ≥ 25 was found among patients on DTG-based regimens (34.5%) (30/86) than on ATV/r-based regimens of ART (23%) (20/86). The detailed description of these and other variables were displayed by the following Table 2.

### 3.3. The Mean Values of AIP and hsCRP Among Study Groups

According to the our study results displayed in Table 3, the higher mean levels of AIP were observed among patients treated by DTG-based regimens (5.4) than in ATV/r-based regimens (4.7) (*p*=0.02). But insignificant differences was found regarding hsCRP levels (*p*=0.18).

### 3.4. Correlation of AIP and CD4 Cell Counts

**Figure 1:**
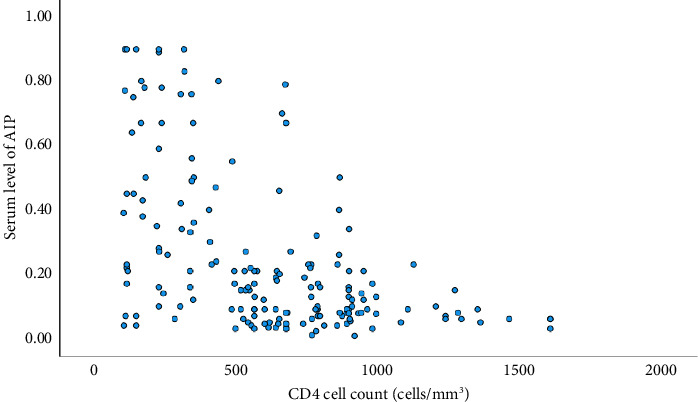
Correlation of AIP and CD4 cell counts.

According to Figure 1, the levels of AIP were correlated negatively with the CD4 cell counts of the participants, and it was observed to be statistically significant (R value of −0.46).

### 3.5. Correlation of hsCRP and CD4 Cell Counts

**Figure 2:**
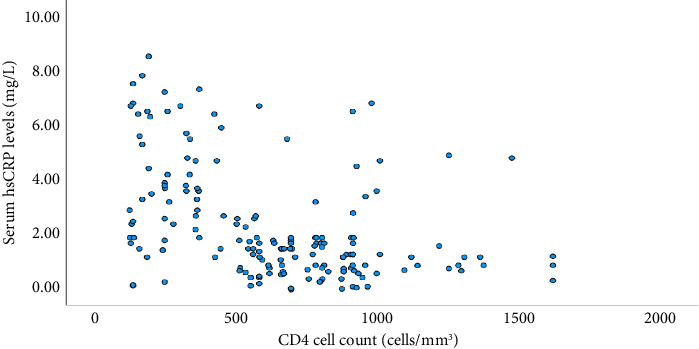
Correlation of hsCRP levels and CD4 cell counts.

The current study reported that a statistically significant negative correlation was found between the serum levels of hsCRP and CD4 cell counts of the participants (R value of −0.46), which is described in Figure 2.

### 3.6. Prevalence of AIP and hsCRP Levels Among Study Groups

As shown by Table 4, the proportions of AIP, 0.1–0.24 and > 0.24 were higher in patients treated by ATV/r-based regimen as compared with DTG-based regimens. Regarding hsCRP levels, the higher levels of hsCRP ≥ 2 mg/L were found among those patients treated by ATV/r-based regimens of ART than those by DTG-based regimens (0.006).

## 4. Discussion

In this study, the levels of AIP and hsCRP in PLWH receiving DTG and ritonavir-boosted ART were investigated, along with their relationship to the CD4 cell counts. As far as we are aware of it, this is the first study which is conducted in Ethiopia; particularly considering recently introduced new ART the medication DTG for HIV therapy.

The result of this study showed that a higher proportion of AIP in the range of 0.1–0.24 which is 44.2% and 31.4%, respectively, as well as > 0.24 which is 39.5% and 9.3%, respectively, was seen among patients on ATV/r-based than on DTG-based regimens of ART. This finding is congruent with previous study findings [22, 28]. Increase release of fatty acid from adipocytes and induction of fatty acid synthesis by the liver were some of the reasons why ATV/r increases the level of AIP [52–54].

The current study also found that the serum levels of hsCRP ≥ 2 mg/L was higher in patients treated by ATV/r- than by DTG-based regimens of AR, with the proportions of 24.4% and 44.2%, respectively. This finding of our study is similar with study findings from San Francisco [44], Boston [55] and study from Ethiopia [30]. Increase activation of nuclear factor kB (NF-kB) signaling and increased expression of proinflammatory cytokines which can elevate inflammatory biomarkers with subsequent augmentation of inflammations were some of the possible ways by which ATV/r increases the serum hsCRP levels [56].

Moreover, this study was also designed to assess the correlation of AIP with CD4 cell counts. In accordance, as it was observed from this study, AIP was negatively correlated with CD4 cell counts which are in agreement with study finding done in Nigeria [57] and Chhattisgarh [40]. An increase in AIP with a decrease in CD4 cell counts was attributed to be due to activation of hepatic synthesis of TG as well as decreased triglyceride clearance from the circulations combined with decrease fatty acid oxidation and, furthermore, increased the activities of endothelial lipase and phospholipase-A2 that can lead to reduction of HDL-C levels by increasing its clearance from the circulations [58–60].

Similarly, our study findings revealed that hsCRP levels and CD4 cell counts of the participants were negatively correlated. This finding is in line with previous a study findings from Chhattisgarh [40]. However, our finding was in conflict with a study from Brazil [32]. The possible reasons for this might be the participant in the Brazil study included ART-naïve; however, our study does not include ART-naïve patients and also most of the patients were treated by NRTI in Brazil study. On the contrary, in the current study, an equal number of patients took DTG as well as ATV/r-based regimens of ART. In addition, sociodemographic and sample size differences might contribute to this in the conflict of findings of the two studies. Negative correlation of hsCRP with CD4 cell counts was possibly due to increase immune activation and viral loads-mediated increase destruction of CD4-T cells and concurrent opportunistic infections that increase hsCRP levels [61].

### 4.1. Limitations and Strength

Concerning to strength, this was the first study in Ethiopia that attempted to assess the effects of DTG- and ATV/r-based regimens on AIP and hsCRP levels among PLWH, hence ultimately adding to the already available limited data. In the face of this strength, this study has several weaknesses. Since it is a cross-sectional study, we cannot associate causal relationships between the factors and outcomes under study. In addition, the study sample size was small; thus, it is difficult to generalize the findings to larger populations.

## 5. Conclusions and Recommendations

In summary, this study found a higher proportion of AIP and hsCRP among PLWH taking ATV/r-based than DTG-based regimen of ART. AIP and hsCRP levels were negatively correlated with CD4 cell counts. This indicates that AIP and hsCRP levels may be important markers to indicate prognosis of HIV and its progressions. They can also indicate response to treatment for PLWH. Relative to CD4 cell counts, AIP and hsCRP tests are cost-effective and affordable for many patients living in developing countries like Ethiopia. So, even if AIP and hsCRP levels cannot replace CD4 cell counts, they can show the overall state of the patients. Since increase in both AIP and hsCRP markedly increase the risk of having CVD, measuring the baseline AIP as well as hsCRP levels should be part of routine care for PLWH on HAART, particularly for PLWH taking ATV/r-based regimens of ART and those with low CD4 cell counts for early detection and prevention of the risk of developing CVD.

Policymakers should take these factors into account when developing public health initiatives on ART side-effects management and when strengthening on going noncommunicable disease reduction programs. We propose that researchers conduct a prospective cohort study with a larger sample size to determine the exact effects of DTG and ATV/r on AIP and hsCRP levels. Lastly, we suggest a comparative analysis of DTG and ATV/r containing ART in ART-naïve, HIV-positive patients.

## Figures and Tables

**Table 1:** Sociodemographic characteristics of study participants.

Variables	Category	Exposure category		*p*-value
		DTG *n* (%)	ATV/r *n* (%)	
Age	18–39	41 (47.7)	43 (50)	
	≥ 40	45 (52.3)	43 (50)	0.8

Gender	Male	29 (33.7)	26 (29.2)	
	Female	57 (66.3)	60 (69.8)	0.08

Religion	Muslim	25 (29.1)	29 (33.7)	
	Orthodox	28 (32.6)	27 (31.4)	0.8
	Others	33 (38.4)	30 (34.9)	

Ethnicity	Oromo	41 (47.7)	48 (55.8)	0.5
	Amhara	19 (22.1)	18 (20.9)	
	Others	26 (30.2)	20 (23.3)	

Marital status	Married	59 (77.9)	67 (68.6)	
	Unmarried	27 (31.4)	19 (22.1)	0.1

Educational status	Educated	81 (94.2)	80 (93)	
	Uneducated	5 (7)	6 (5.8)	0.8

Occupational status	Employed	26 (30.2)	21 (24.4)	
	Unemployed	60 (69.8)	65 (65.6)	0.4

Income (Birr/month)	< 5000	44 (51.2)	50 (58.1)	
	≥ 5000	42 (48.8)	36 (41.9)	0.35

Residence	Urban	66 (76.7)	66 (76.7)	
	Rural	20 (23.3)	20 (23.3)	0.6

Smoking status	Yes	0 (0)	1 (1.6)	
	No	64 (100)	63 (98.4)	0.32

Alcohol use practice	Yes	40 (46.5)	47 (54.7)	
	No	46 (53.5)	39 (45.3)	0.28

Physical activity	Yes	68 (67.6)	68 (79.9)	
	No	28 (32.6)	18 (20.9)	0.8

*Note:* ATV/r = ritonavir-boosted atazanavir, *n* (%) = number.

Abbreviation: DTG = dolutegravir.

**Table 2:** Clinical characteristics of the study participants.

Baseline variables	Category	Exposure category		*p* value
		DTG-based ART	ATV/r-based ART	
CD4 cell count (cells/mm^3^) [mean ± SD]	NA	615 ± 336	543 ± 386	0.19

Viral load (copies/mL) [*n* (%)]	≤ 1000	78 (90.7)	76 (88.4)	
	> 1000	8 (9.3)	10 (11.6)	0.6

Duration of HIV (years) [*n* (%)]	≤ 5	46 (53.5)	49 (57)	
	> 5	40 (46.5)	37 (43)	0.6

Duration of ART (month) [*n* (%)]	≤ 24	36 (41.9)	44 (52.3)	
	> 24	50 (58.1)	41 (47.7)	0.54

WHO clinical staging [*n* (%)]	I	21 (24.4)	25 (29.1)	
	II	33 (38.2)	26 (30.2)	
	III	32 (37.2)	35 (40.7)	0.5
	IV	0	0	

BMI (kg/m^2^) [*n* (%)]	< 25	56 (65.1)	66 (76.7)	
	≥ 25	30 (34.9)	20 (23.3)	0.093

WC (cm) [*n* (%)]	< cut-off	43 (50)	42 (48.8)	
	≥ cut-off	43 (50)	44 (51.2)	0.9

WHR [*n* (%)]	< cut-off	41 (47.7)	44 (51.2)	
	≥ cut-off	45 (52.3)	42 (48.8)	0.6

*Note:* Student's *t*-test was used for continuous variables presented by mean ± SD, Mann–Whitney test for continuous variables presented by median (IQR), and Chi square tests for categorical variables. ATV/r = ritonavir-boosted atazanavir, ART = highly active antiretroviral therapy, *n* (%) = number and percent.

Abbreviations: BMI = body mass index, DTG = dolutegravir, IQR = interquartile range, kg/m^2^ = kilogram per meter square, NA = not applicable, SD = standard deviation, WC = waist circumference, WHR = waist-hip ratio.

**Table 3:** Mean difference between the study groups.

Variables	Exposure category		*p* value
	ATV/r-based regimen	DTG-based regimen	
AIP (mean ± SD)	4.7 ± 1.7	5.38 ± 2.1	0.02
hsCRP (mean ± SD)	2.9 ± 2.8	2.3 ± 2.5	0.18

*Note:* ± = plus or minus, ATV/r = ritonavir-boosted atazanavir.

Abbreviations: AIP = atherogenic index of plasma, DTG = dolutegravir, hsCRP = high sensitivity C-reactive protein, SD = standard deviation.

**Table 4:** Categorical comparison of AIP and hsCRP levels among the study groups.

Variables	Category	Exposure category		*p* values
		DTG-based regimens	ATV/r-based regimens	
AIP	< 0.1	44 (51.2)	21 (24.4)	0.01
	0.1–0.24	34 (39.5)	38 (44.2)	
	> 0.24	8 (9.3)	27 (31.4)	

hsCRP	< 2 mg/L	65 (75.6)	48 (55.8)	0.006
	≥ 2 mg/L	21 (24.4)	38 (44.2)	

*Note:* ATV/r = ritonavir-boosted atazanavir.

Abbreviations: AIP = atherogenic index of plasma, DTG = dolutegravir, hsCRP = high sensitivity C-reactive protein.

## Data Availability

All the necessary materials can be found in the text. Due to the privacy policy, confidential data materials could be obtained from the corresponding author upon request.

## Nomenclature

AIP: Atherogenic index of plasma
ART: Antiretroviral therapy
ASCVD: Atherosclerotic cardiovascular disease
ATV/r: Ritonavir-boosted atazanavir
BMI: Body mass index
CD4: Cluster of differentiation-4
CVD: Cardiovascular disease
DTG: Dolutegravir
EFV: Efavirenz
hsCRP: High-sensitivity C-reactive protein
HAART: Highly active antiretroviral therapy
HIV: Human immuno deficiency virus
INSTI: Integrase strand transfer inhibitor
IQR: Interquartile range
JUMC: Jimma University Medical Centre
NF-kB: Nuclear factor kappa B
SPSS: Statistical package for social sciences
TDF: Tenofovir
